# FAM3B promotes progression of oesophageal carcinoma via regulating the AKT–MDM2–p53 signalling axis and the epithelial‐mesenchymal transition

**DOI:** 10.1111/jcmm.14040

**Published:** 2018-12-18

**Authors:** Song‐Lin He, Wen‐Ping Wang, Yu‐Sang Yang, En‐Min Li, Li‐Yan Xu, Long‐Qi Chen

**Affiliations:** ^1^ Department of Thoracic Surgery West China Hospital of Sichuan University Chengdu China; ^2^ North Sichuan Medical College Nanchong China; ^3^ The Key Laboratory of Molecular Biology for High Cancer Incidence Coastal Chaoshan Area Shantou University Medical College Shantou China; ^4^ Department of Biochemistry and Molecular Biology Shantou University Medical College Shantou China; ^5^ Institute of Oncologic Pathology Shantou University Medical College Shantou China

**Keywords:** AKT, apoptosis, epithelial‐mesenchymal transition, esophageal carcinoma, FAM3B, invasion, MDM2, p53

## Abstract

FAM3B has been suggested to play important roles in the progression of many cancers, such as gastric, oral, colon and prostate cancer. However, little is known about the role of FAM3B in human esophageal squamous cell carcinoma (ESCC). In the present study, we found that FAM3B expression was higher in ESCC tissues than in adjacent normal tissues. Using quantitative real‐time polymerase chain reaction, we found similar results in cell lines. FAM3B expression was significantly related to T/TNM stage. Importantly, Kaplan–Meier analysis revealed that a high expression level of FAM3B predicted a poor outcome for ESCC patients. Overexpression of FAM3B inhibits ESCC cell death, increases oesophageal tumour growth in xenografted nude mice, and promotes ESCC cell migration and invasion. Further studies confirmed that FAM3B regulates the AKT–MDM2–p53 pathway and two core epithelial‐to‐mesenchymal transition process markers, Snail and E‐cadherin. Our results provide new insights into the role of FAM3B in the progression of ESCC and suggest that FAM3B may be a promising molecular target and diagnostic marker for ESCC.

## 1 INTRODUCTION

Esophageal squamous cell carcinoma (ESCC) is the leading cause of cancer death in China.^1^, ^2^, ^3^ According to the 2015 Chinese Cancer Statistics, the morbidity of oesophageal cancer ranked 5th and its mortality ranked 4th among all kinds of malignant tumours.^4^ Much effort has focused on improving tumour resectability and long‐term locoregional control, but oesophageal cancer is still characterized by poor 5‐year overall survival (OS), which rarely exceeds 40%.^5^, ^6^ In addition to advances in surgical techniques and multimodal therapies to improve survival, research in the past decade has produced effective molecular targeted treatments for cancer therapy.^7^, ^8^ Compared with lung cancer and colorectal cancer, the current targeted treatment for oesophageal carcinoma is still at the grouping stage, and no definite sensitive molecular markers and mechanisms have been identified.^8^, ^9^, ^10^

Family with sequence similarity 3 (FAM3) is a cytokine‐like gene family identified in 2002 through computational genomic searching for novel cytokines using structure‐based homology methods. This family includes four genes: *FAM3A*, *FAM3B*, *FAM3C* and *FAM3D*.^11^ Over the past decade, intensive studies have suggested that members of the FAM3 gene family may play an important role in the development of a variety of major diseases, including diabetes and cancer.^12^, ^13^, ^14^, ^15^

Of the four family members, FAM3B has been studied the most.^16^ FAM3B (also called PANDER) was originally identified in the endocrine pancreas and is also expressed in the small intestine, stomach and colon. In addition to identifying its role in glycolipid metabolism, research on FAM3B focuses on its biological role in tumour progression. In 2008, Huang et  al^17^ first reported that the expression level of FAM3B was lower in human gastric cancer tissues than in adjacent normal tissues and that its lower mRNA level was associated with deeper tumour invasion of gastric cancer tissues. These findings suggested that abnormal expression of FAM3B might be involved in cancer initiation and progression. Since then, the regulatory role of FAM3B in the progression of other tumours, including oral squamous cell carcinoma and colon and prostate cancer, have been reported.^18^, ^19^, ^20^ In addition, another FAM3 family member, FAM3C, was reported to be closely involved in the development of oesophageal cancer.^21^ These studies have increased interest in exploring whether FAM3B also plays an important role in the progression of oesophageal cancer.

In this study, we demonstrate for the first time that expression of FAM3B is significantly up‐regulated in ESCC. Its biological function in ESCC appears to be to inhibit cell death and promote cell migration and invasion rather than regulate the cell cycle. We explored further the mechanisms involved in FAM3B‐induced progression of ESCC, and we confirmed the regulatory relationship between FAM3B and the downstream signalling pathway. Our results suggest that FAM3B may be a new therapeutic target and diagnostic marker for ESCC.

## 2 MATERIALS AND METHODS

### 2.1 Tissue specimens and clinicopathological data collection

Forty paraffin‐embedded ESCC tumour specimens and their adjacent tissues were included. The pathology of these specimens had been diagnosed at West China Hospital of Sichuan University in 2010‐2012. The clinicopathological classification of all patients was confirmed according to the criteria of the Union for International Cancer Control, which is non‐governmental organization that exists to help the global health community accelerate the fight against cancer. The clinicopathological information for the samples included age, gender, pathological grade, tumour stage, lymph node metastatic status, and TNM classification. Patients were closely followed up every 3 months during the first 2 postoperative years, and every 6 months thereafter. OS time was calculated from the date of the initial surgery to death. The study was performed with the approval of the hospital ethics committee, and written informed consent was obtained from all patients.

### 2.2 Tissue microarray and immunohistochemistry

Constructed tissue microarrays (TMAs) contained 40 pairs of ESCC tissue samples and their corresponding adjacent nontumour tissues, as described previously. The TMA sections were first dewaxed and then washed with phosphate buffer saline (PBS) and incubated with 3% H_2_O_2_ for 10 minutes. Antigen retrieval was performed by heating the sections from 65°C to 95°C for 20 minutes in a pressure cooker. The temperature was then decreased to room temperature, and the sections were blocked with 5% normal rabbit serum for 1 hour and incubated with rabbit anti‐human FAM3B polyclonal antibody (Abcam, Cambridge, UK) overnight at 4°C. The slides were washed and incubated with biotinylated secondary antibody conjugated with haptoglobin related protein (HRP, 1:500) for 1 hour at room temperature. The expression of FAM3B was scored as high or low independently by two experienced pathologists.

### 2.3 Cell culture

Human ESCC cell lines (ECA109, KYSE150, KYSE9706 and TE‐1) and oesophageal epithelial cells (HET‐1A) were kindly provided by the State Key Laboratory of Biotherapy of Sichuan University. The cells were cultured in RMPI 1640 medium (Hyclone, USA) with 10% fetal bovine serum (FBS; Gibco, USA) and penicillin, and were maintained in a humidified incubator at 37°C with 5% CO_2_. The cells were passaged when the cell density reached 80%‐90%, and the medium was replaced every 2‐3 days.

### 2.4 Real‐time PCR

Total RNA was isolated from tissue specimens and cells using the Axygen Mini Kit (Corning, Union City, CA, USA) according to the manufacturer’s protocol. The extracted RNA was then reverse transcribed to cDNA using a PrimeScript™ RT Reagent Kit with gDNA Eraser (Takara, Tokyo, Japan) following the manufacturer’s instruction. The cDNA template was mixed with the gene primers of interest and SYBR Green 2X mixture (Bio‐Rad, Hercules, CA, USA). Quantitative real‐time polymerase chain reaction analysis (q‐RT‐PCR) of the reaction mixtures was performed using a Bio‐Rad CFX96 Touch (Bio‐Rad). All primer sequences used are listed in Table 1. The expression level of the target mRNA was calculated by the 2^–∆∆CT^ method. Relative quantification was performed with glyceraldehyde‐3‐phosphate dehydrogenase (GAPDH) transcript as an endogenous housekeeping control.

**Table 1:** Primers used in this study

Primer	Sequence (5′‐3′)
GAPDH‐F	CAGGAGGCATTGCTGATGAT
GAPDH‐R	GAAGGCTGGGGCTCATTT
FAM3B‐F	CCTCCTTQTQTQCCTQQTAT
FAM3B‐R	QQACTQQAQCTTTQAQQACA

### 2.5 Colony‐formation assay

A colony‐formation assay was performed to detect cell tumorigenic capability. The cells were seeded in six‐well plates (500 cells/well), and the medium was changed every 5 days. The plates were maintained under standard breeding conditions for 14 days, fixed with absolute methanol for 15 minutes, and stained with crystal violet for 20 minutes. Colony counts were based on macroscopic biocenosis.

### 2.6 Migration and invasion assays

Millicell^®^ Transwell chambers with a pore size of 8 μm (Millipore, MA, USA) were used with or without Matrigel (BD Biosciences, Franklin Lakes, NJ, USA) to quantify cell migration and invasion. For the invasion assay, the Transwell chamber was precoated with 0.6 mL of a mixture of Matrigel and RMPI 1640 (1:5, v/v), and was placed into a 24‐well plate and kept for 1 hour in an incubator at 37°C. In both Transwell assays, 4 × 10^4^ tumour cells in 0.25 mL of serum‐free medium were plated into the upper chamber, and 0.6 mL of the medium containing 10% FBS was added to the lower chamber. After 48 hours of incubation, cells on the upper surface of the Millicell chambers, which are noninvasive cells, were scraped with a cotton swab. Tumour cells on the bottom surface, the transmembrane cells, were fixed in 90% ethanol for 20 minutes and stained with 0.1% crystal violet for 15 minutes.

### 2.7 Cell cycle assays

In cell cycle assay, cells were first transfected for 48 hours and then fixed with 75% ethanol and stained with 500 μL of PBS containing 50 μg/mL propidium iodide and 0.1% TritonX‐100 in the dark at 4°C. All samples were analysed by flow cytometry, and the data were processed using Cell Quest software. The apoptosis rates of ESCC cell lines were measured by flow cytometry 48 hours after transfection. DNA fragmentation assays were conducted using AnnexinV‐FITC Apoptosis Detection Kit (BD Biosciences) according to the manufacturer’s protocol.

### 2.8 Protein extraction and Western blot analysis

Cells were lysed in ice‐cold lysis buffer containing fresh protease inhibitor. Cellular lysate extracts were scraped from the wells, collected in Eppendorf tubes, and centrifuged at 12 000 *g* for 10 minutes at 4°C. The total cell proteins were obtained and quantified using a NanoDrop^TM^ 2000/2000c spectrophotometer (Thermo Fisher Scientific, Waltham, MA, USA). Equal amounts of proteins were separated by sodium dodecyl sulfate‐polyacrylamide gel electrophoresis, and the separate proteins were transferred to a PVDF membrane. The membrane was blocked with 5% fat‐free milk at 37°C for 1 hour and then incubated with the homologous primary antibodies (Abcam) overnight at 4°C. The membranes were rinsed, incubated in peroxidase‐conjugated secondary antibody at 37°C for 30 minutes, and analysed using a chemiluminescence system (Amersham Biosciences, UK). The stained bands were scanned and filed, and the pixel intensity was analysed using Alpha processing software (Alpha Innotech, Chengdu, china).

### 2.9 Lentivirus packaging and transduction

The recombinant vectors were first constructed by cloning full‐length human FAM3B cDNA into the pEB‐GFP (T2A) PURO vector using a Clon Express II One Step cloning kit (Vazyme, Nanjing, China) according to the manufacturer’s protocol and then transformed into Stbl3 competent cells. The positive clones identified by colony PCR were confirmed by sequencing. The lentiviral vectors carrying FAM3B were successfully constructed. A mixture of the lentiviral plasmids, two auxiliary plasmids (psPAX2 and PMD2.G) and the transfection reagents (Fitgene, Guangzhou, China) were added into a culture dish for HEK293T cells. The medium was replaced with Dulbecco’s modified Eagle’s medium (DMEM) containing 10% FBS 4‐6 hours after transfection. The medium containing the viral particles was collected 48 hours after transfection, centrifuged at low speed (112 *g* for 5 minutes), and filtered through a 0.45 μm filter. The viruses were concentrated by high‐speed refrigerated centrifugation (25 000 *g* for 2 hours). The FAM3B‐overexpressing plasmid‐transfected HEK293 T cells produced the lentiviral particles with a titer of 3 × 10^8^ TU/mL, which showed the success of the recombinant lentivirus package. The FAM3B‐overexpressing or FAM3B‐negative control (NC) lentiviruses were then transfected into the ECA109 cell line using Lipofectamine 2000 (Thermo Fisher Scientific) according to the manufacturer’s protocol.

### 2.10 Tumour formation in nude mice

Animal experiments were conducted in the State Key Laboratory of Biotherapy of Sichuan University according to the protocols approved by laboratory animal center of Sichuan University. The in vivo tumour‐promoting ability of FAM3B was studied using a tumorigenesis model in nude mice. Five male mice aged 5 weeks were purchased from Beijing Vitonglihua Bio‐technology Co. Ltd (China). Five million FAM3B vector ECA109 cells in 100 μL of PBS and the same number of control cells were injected subcutaneously into the left and right armpit of BALB/c nude mice, respectively. Tumour volume (V) was monitored twice a week for 4 weeks. V was calculated from measurement of the length (L) and width (W) of xenograft tumours and calculated using the formula V = L × W^2^/2. After 4 weeks, the mice were sacrificed and tumour tissues were removed. The protein was extracted from the tumour tissues and used for Western blot analysis.

### 2.11 Plasmid construction and RNA interference

A plasmid expressing FAM3B was purchased from FitGene (Guangzhou, China). The siRNA sequence targeting human FAM3B was designed and synthesized by Ribobio (Guangzhou, China). An empty plasmid or scrambled siRNA served as a NC. TE‐1 and ECA109 cells were seeded into six‐well plates in DMEM containing 10% FBS. Once the cells were 50%‐60% confluent, the medium was switched to Opti‐MEM Reduced Serum Medium (Gibco). To optimize the concentration of the transfection reagent, different concentrations of the FAM3B vector, FAM3B siRNA, or the NC were transfected into the cells using Lipofectamine 2000 (Thermo Fisher Scientific) according to the manufacturer’s protocol. The transfection efficiency was determined by extracting total RNA or protein 24 or 48 hours after transfection and measuring the relative expression of mRNA or protein with qRT‐PCR or Western blot analysis, respectively.

### 2.12 Statistical analyses

Each sample was assayed in triplicate, and each experiment was repeated three times. Chi‐squared test or two‐way ANOVA was used to compare the means of independent samples. Differences between FAM3B levels in tumour tissues and adjacent non‐tumour tissues were analysed using the Wilcoxon matched‐pairs test. The survival rates in relation to FAM3B expression were estimated using the Kaplan–Meier method, and the log‐rank test was used to compare Kaplan–Meier survival curves. Statistical analyses were performed using SPSS for Windows v. 16.0 (SPSS Inc, Chicago, IL, USA). *P*‐values <0.05 were considered to be significant.

## 3 RESULTS

### 3.1 FAM3B was overexpressed in ESCC tumour specimens and cell lines

**Figure 1:**
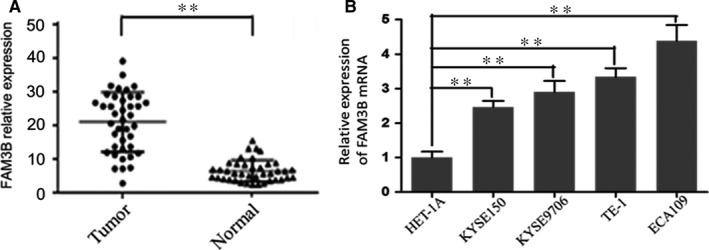
Up‐regulation of FAM3B mRNA expressions in ESCC tissue samples and ESCC cell lines. The relative expression of FAM3B mRNA was analysed by qRT‐PCR. GAPDH was used as an internal control. A, 40 surgically resected tumour specimens and their adjacent tissues. B, Human ESCC cell lines (KYSE150, KYSE9706, TE‐1 and ECA109) and oesophageal epithelial cell line (HET‐1A). **P < 0.001

**Figure 2:**
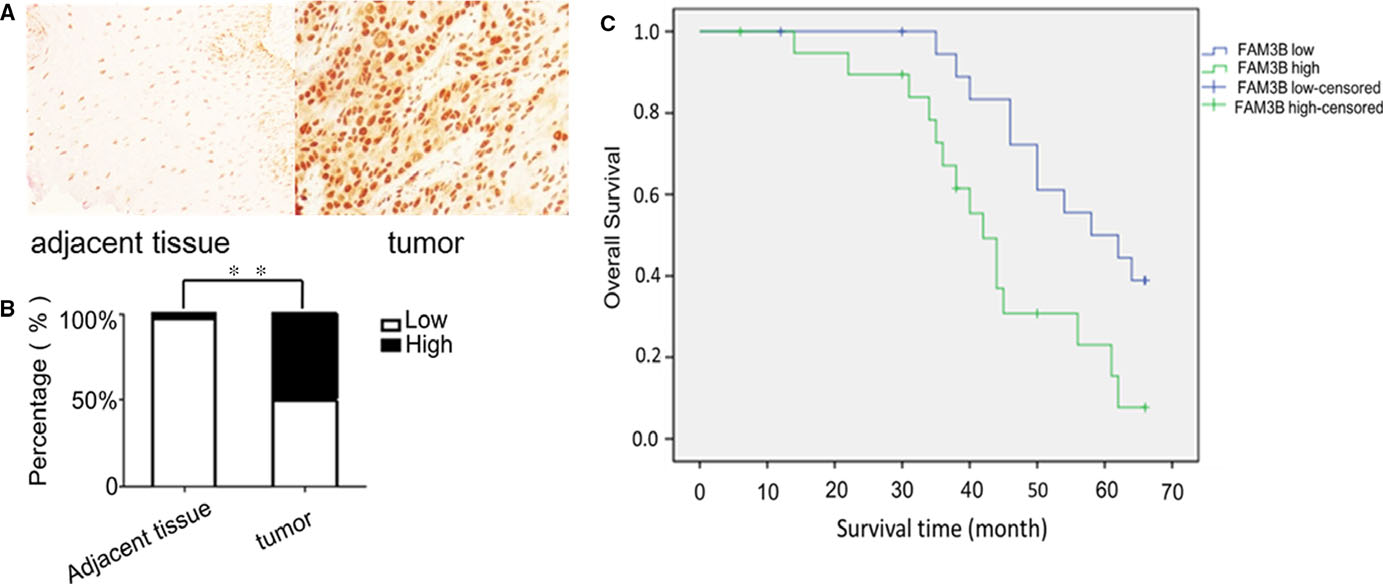
Immunohistochemical analysis of FAM3B in ESCC and its correlation with clinical prognosis. A, Representative results of immunohistochemical analysis of FAM3B in ESCC tissue samples and adjacent normal tissues. Original magnification, ×400. B, Results of immunohistochemical staining in the human tissue array. C, Kaplen–Meier analysis of OS in ESCC patients. Blue, group with low FAM3B expression; green, group with high FAM3B expression. ** *P* < 0.01

To explore FAM3B expression in ESCC, 40 surgical tumour samples of oesophageal carcinoma and their adjacent normal tissue were analysed using qRT‐PCR analysis and immunohistochemistry. The clinicopathological data are shown in Table 2. The qRT‐PCR analysis showed that FAM3B mRNA expression was up‐regulated in 85% (34/40) of the tumour samples compared with matched adjacent normal tissues (*P* < 0.01) (Figure 1A). Similar results for the qRT‐PCR analysis were obtained for four ESCC cell lines (ECA109, KYSE150, KYSE9706 and TE‐1) and the normal oesophageal epithelial cell line HET‐1A. FAM3B mRNA expression was lower in HET‐1A cells than in the four ESCC cell lines. The relative expression levels were 4.38 and 3.34 in ECA109 and TE‐1 cell lines, respectively (Figure 1B). The immunohistochemical results were consistent with the transcription data. That is, FAM3B protein was located primarily in the nucleus of ESCC cells and was expressed at a higher level in tumour tissue than in adjacent normal tissue (Figure 2A,B).

**Table 2:** Clinicopathological features of ESCC patients

Clinical parameter	n
Gender	
Male	23
Female	17
Age, years	
<55	17
≥55	23
Lymph node metastasis	
No	24
Yes	16
Differentiation	
Good	6
Moderate	15
Poor	19
Invasion	
T_1_	5
T_2_	17
T_3_	13
T_4a_	5
TNM stage	
I	7
II_a_	8
II_b_	9
III	16

### 3.2 FAM3B expression is associated with ESCC clinicopathological parameters and prognosis

We explored further whether FAM3B protein expression was significantly related to the clinicopathological parameters of ESCC patients. FAM3B expression was not significantly related to gender, age, differentiation, or lymph node metastasis. By contrast, increased expression of FAM3B in ESCC was significantly associated with invasion and TNM stage (*P* = 0.011 and 0.003, respectively; Table 3). The FAM3B expression measured by immunohistochemistry was used to classify ESCC patients into two groups: a high‐expression group (20 patients) and low‐expression group (20 patients). Their prognosis was measured as OS. The respective average survival and 5‐year survival rates were 58 months and 45% for the low‐expression group and 42 months and 15% for the high‐expression group. Kaplan–Meier survival analysis revealed that patients in the high‐expression group had poorer OS than those in the low‐expression group (*P* < 0.01; Figure 2C).

**Table 3:** Correlations between FAM3B expression and clinicopathological features of oesophageal carcinoma patients

Characteristic	FAM3B expression			*P*‐value
	Low		High	
Gender				
Male	9	14		0.110
Female	11	6		
Age, years				
<60	7	10		0.337
≥60	13	10		
Lymph node metastasis				
No	14	10		0.197
Yes	6	10		
Differentiation				
G1	4	2		0.675
G2	7	8		
G3	9	10		
Invasion				
T_1_+T_2_	15	7		0.011
T_3_+T_4a_	5	13		
TNM stage				
I–II_a_	12	3		0.003
II_b_–III	8	17		

### 3.3 FAM3B promotes ESCC progression in vitro and in vivo

**Figure 3:**
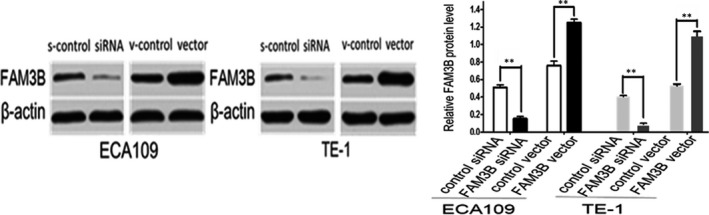
The protein expression level of FAM3B was examined by Western blot analysis after transfection. Representative images and quantification of Western blotting analysis are shown. **P < 0.01

To assess the biological role of FAM3B in ESCC progression, we performed a colony‐formation assay, migration (invasion) assay, flow cytometry assays and animal experiments in nude mice. First, we transfected a siRNA that down‐regulated the expression of FAM3B or a plasmid that up‐regulated the expression of FAM3B into ECA109 and TE‐1 cells, and then measured the transfection efficiency after FAM3B knockdown or overexpression by Western blot analysis (Figure 3).

**Figure 4:**
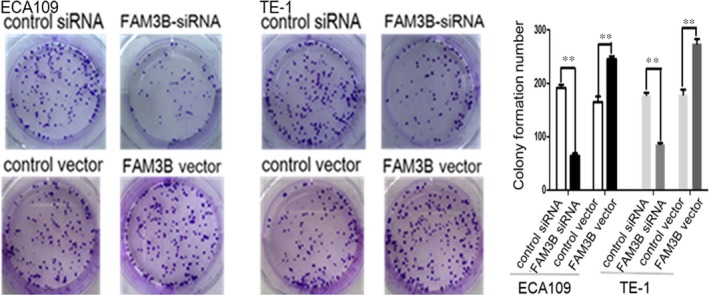
FAM3B promotes ESCC cell tumorigenic capability. Representative images of colony formation and its quantification showing the colony numbers after FAM3B knockdown or overexpression

**Figure 5:**
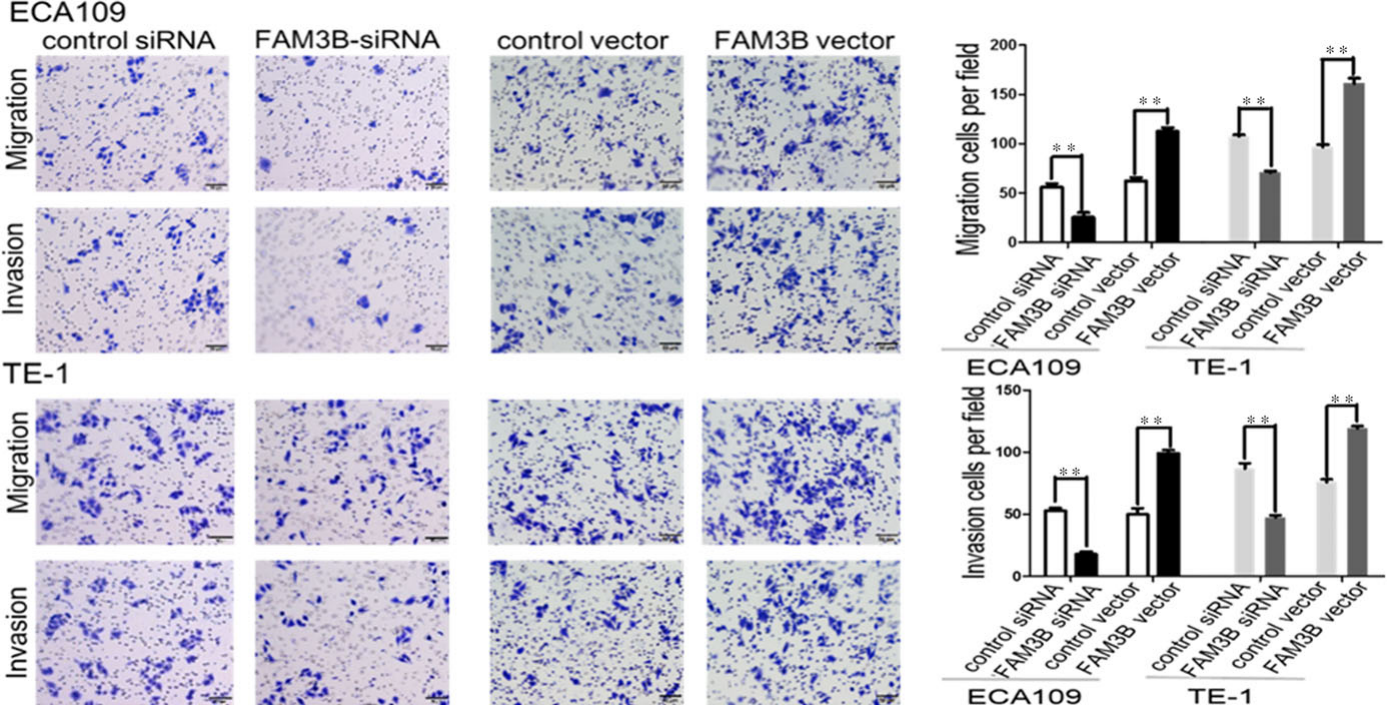
FAM3B promotes ESCC cell migration and invasion. Representative images of in vitro migration and invasion assays in the Transwell system and their quantification showing the number of migration and invasive cell after FAM3B knockdown or overexpression

**Figure 6:**
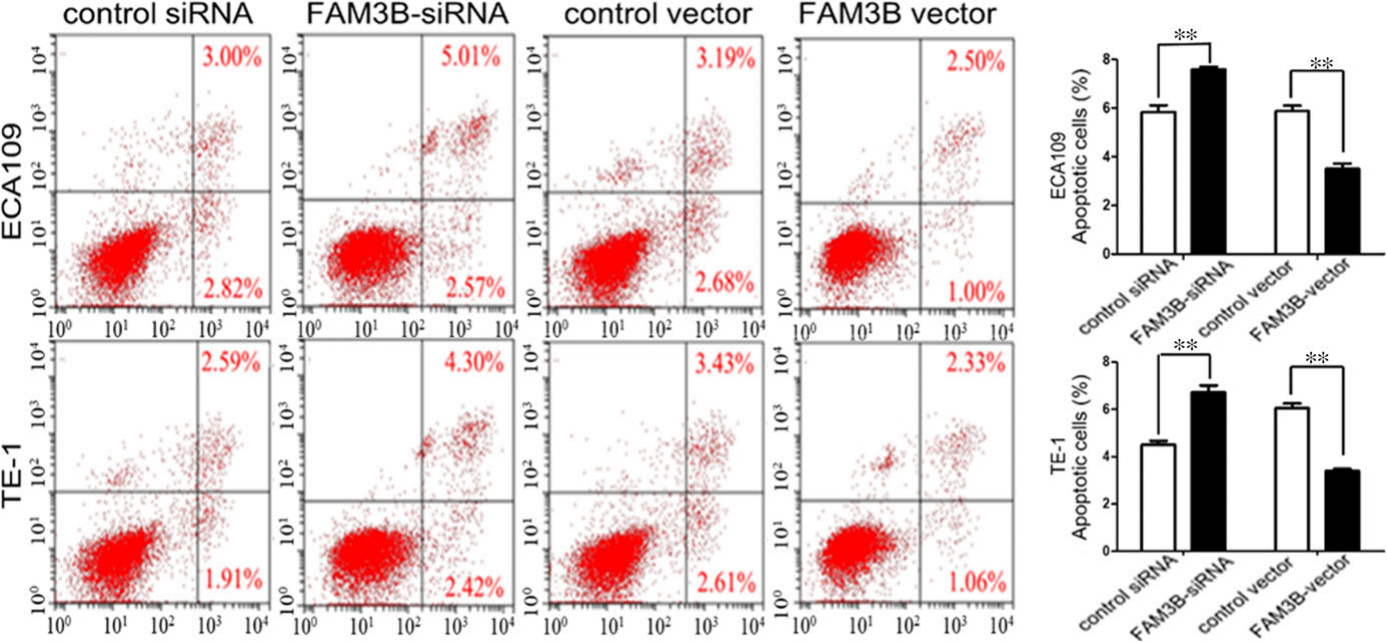
FAM3B affects ESCC cell death. Representative images of flow cytometric analysis of DNA fragmentation and its quantification showing apoptotic rates after FAM3B knockdown or overexpression

The colony‐formation assay showed that FAM3B silencing significantly inhibited the tumorigenic capability of ESCC cells. Conversely, overexpression of FAM3B induced the opposite effect (Figure 4). We next examined the effect of FAM3B on cell migration and invasion. The migration and invasion abilities were markedly reduced in FAM3B‐silenced ESCC cells compared with cells transfected with control siRNA. By contrast, increased FAM3B expression increased the migration and invasion abilities of ESCC cells (Figure 5). Flow cytometric analysis of DNA fragmentation showed that the apoptotic rates were reduced by up‐regulated expression of FAM3B but increased by down‐regulated expression of FAM3B (Figure 6).

**Figure 7:**
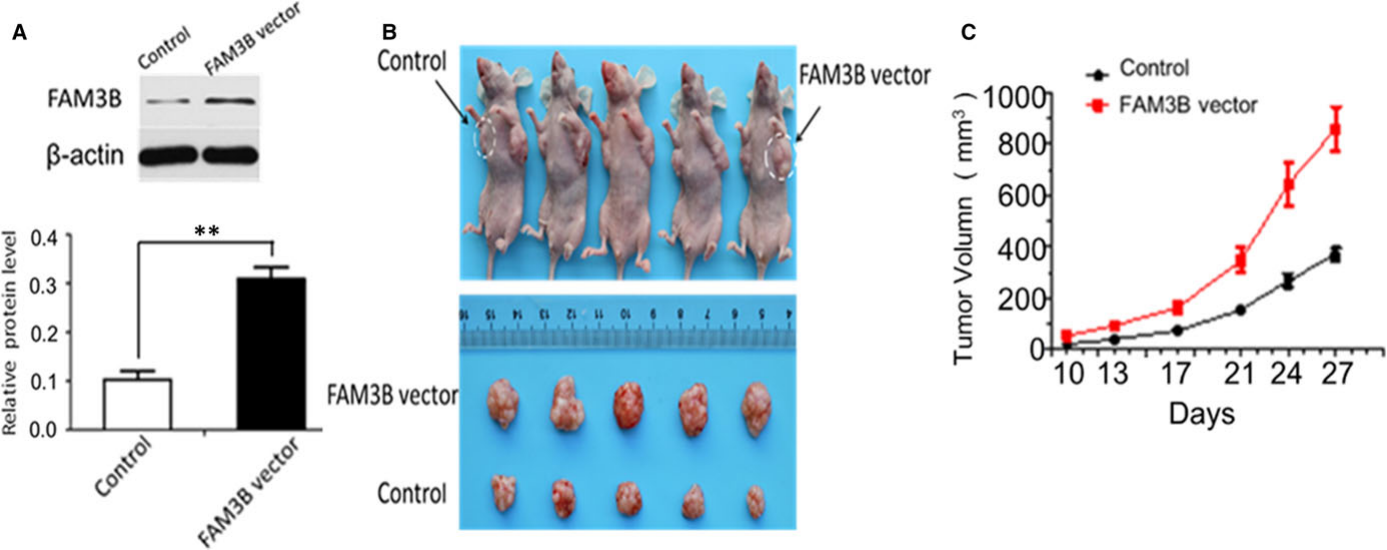
Overexpression of FAM3B accelerates the tumour growth in vivo. A, FAM3B transfection efficiency in ECA109 cell was measured by Western blot. B, The experimental mouse model was constructed by inoculating ECA109 cells transfected with the control vector or FAM3B vector, A representative image shows a larger tumour size in the left armpit (FAM3B vector group) compared with the right armpit (control group). C, A growth curve for the xenograft tumours was drawn. **P < 0.01

To explore further the effect of FAM3B in vivo, we established stably expressed FAM3B using ECA109 cell line, and FAM3B transfection efficiency was measured by Western blot (Figure 7A). ECA109 cells that stably overexpressed FAM3B were implanted into the left armpit and control cells into the right armpit of nude mice. Four weeks after injection, tumours were visible in both armpits of all five mice. As seen in Figure 7B and C, tumours removed from the left armpit were markedly larger in volume than those taken from the right side (*P* < 0.01), which suggested that FAM3B increased tumour growth in vivo mouse model.

### 3.4 FAM3B promotes ESCC progression through regulation of the AKT–MDM2–p53 pathway and epithelial–mesenchymal transition

**Figure 8:**
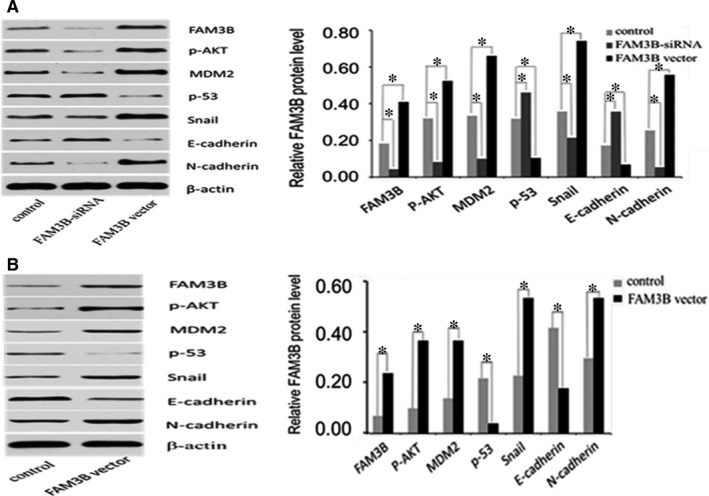
FAM3B promotes ESCC progression by the regulation of AKT–MDM2–p53 signalling pathways and the epithelial–mesenchymal transition. A, The protein expression of genes in ECA109 cells is shown in Western blot images (left) and quantified (right). B, The protein expression of genes in the tumours from nude mice is shown in Western blot images (left) and quantified (right)

To examine whether the AKT–MDM2–p53 pathway and epithelial–mesenchymal transition (EMT) are involved in FAM3B‐induced tumour progression in ESCC, we used Western blot analysis to assess the protein expression of crucial members (p‐AKT, MDM2, p53, Snail, E‐cadherin and N‐cadherin) of the signalling pathways. As shown in the experiments with FAM3B knockdown in ECA109 cells, expression of p‐AKT, MDM2, Snail and N‐cadherin was significantly decreased at the protein level, whereas expression of p53 and E‐cadherin were markedly increased. Overexpression of FAM3B in ECA109 cells produced the opposite effects (Figure 8A). A similar result was also found by analyzing the protein expression in the tumours formed after xenograft of ECA109 cells (Figure 8B). Taken together, these findings support the notion that the ability of FAM3B to promote ESCC progression is, at least in part, dependent on regulation of the AKT–MDM2–p53 pathway and the EMT.

## 4 DISCUSSION

Although several molecular markers closely associated with tumour progression have been recognized, more sensitive markers are needed because of the poor prognosis of ESCC patients. FAM3B is expressed in many normal human tissues, including the pancreas, small intestine, stomach and oesophagus. Initially, FAM3B was confirmed to be a cytokine that triggers cell apoptosis and was therefore assumed to be a potential tumour marker.^11^, ^22^ Until now, a few studies have provided evidence that FAM3B is associated with tumour progression. The study found that elevation of a nonsecretory form of FAM3B (FAM3B‐258) increases migration and invasion of colon cancer cells by up‐regulating Slug expression, which in turn represses the expression of E‐cadherin.^19^ Recent research has shown that increased expression of FAM3B inhibits cell death and plays an important role in promoting the development and progression of prostate cancer,^20^ which is consistent with the roles of FAM3B in colon cancer. By contrast, Kim et  al previously reported that overexpression of FAM3B leads to the inhibition of tumour growth and reduction of the single‐cell colony‐forming activity and tumorigenicity of gastric cancer cells.^23^ Shiiba et  al^18^ recently reported that FAM3B expression in oral squamous cell carcinoma is significantly down‐regulated compared with normal oral tissues. These findings provide evidence that the expression status of FAM3B in cancer cells depends largely on the type of tumour.

In this study, we found that the FAM3B expression level was higher in ESCC tissues than in adjacent normal tissues. Using qRT‐PCR, we obtained similar results in cell lines. We also found a significant association between FAM3B expression level and T/TNM stage. More importantly, Kaplan–Meier analysis revealed that ESCC patients with a high FAM3B expression level had a significantly shorter survival time compared with ESCC patients with low FAM3B expression. In colony‐formation assay, the tumorigenic capability of oesophageal carcinoma cells was increased by FAM3B overexpression but inhibited by FAM3B underexpression. We next detected DNA fragmentation by flow cytometry, and found that apoptotic rates were reduced by up‐regulated expression of FAM3B but increased by down‐regulated expression of FAM3B. Apoptosis assays demonstrated that the increased formation of colonies might be the result of the anti‐apoptotic effect of FAM3B on ESCC cell lines. In addition, cells transfected with the FAM3B vector showed increased migration and invasion capabilities, whereas cells transfected with FAM3B siRNA showed impaired migration and invasion. These findings suggest that FAM3B may function as a tumour progression promoter that contributes to promote ESCC progression, and that it may be a potential biomarker for predicting the prognosis of ESCC patients.

The essential question relates to the mechanism to explain how dysregulated FAM3B expression inhibits ESCC cell death and promotes ESCC cell migration and invasion. AKT is an inhibitor of apoptosis and contributes to cancer progression.^24^ The phosphorylated form of AKT (p‐AKT) has been shown to negatively regulate p53, which is encoded by the homologous gene TP53 and is recognized as a crucial tumour suppressor in multicellular organisms. In this process of AKT‐mediated reduction of p53, the phosphorylation of MDM2 induced by p‐AKT triggers the translocation of MDM2 from the cytoplasm into the nucleus, where nuclear MDM2 degrades p53 protein and down‐regulates p53 function, which helps cells to counterbalance p53‐mediated apoptosis.^25^ MDM2 can also be regulated by p53‐induced negative feedback. p53 can indirectly inhibit the role of AKT by up‐regulating PTEN, an endogenous phosphatase that targets AKT dephosphorylation.^26^

The AKT–MDM2–p53 signalling axis has a profound effect on cell apoptosis. This signalling pathway is involved in the development and progression of many cancers, including prostate cancer,^27^ bladder cancer,^28^ ovarian cancer^29^ and breast cancer.^30^ However, the regulators of the AKT–MDM2–p53 pathway in ESCC are poorly understood. A recent study demonstrated that FAM3B‐induced cell apoptosis is physically associated with p53,^31^ which suggests that FAM3B may play an important role in regulating cell growth through a p53‐dependent pathway. To explore whether the AKT–MDM2–p53 signalling axis is also a downstream target of FAM3B in ESCC, we used Western blot analysis to show that upregulation of FAM3B increased the expression of p‐AKT and MDM2, and decreased the expression of p53, and that silencing of FAM3B produced the opposite effects. These findings suggest that FAM3B may weaken the tumour‐suppressive effect of p53 by activation of the AKT–MDM2 pathway in ESCC.

It is imperative to understand the mechanism controlling how up‐regulation of FAM3B in ESCC increases cell migration and invasion, which is a major reason for the poor prognosis of ESCC patients. Previous studies have shown that the EMT, through which epithelial cells gain migratory and invasive properties to become mesenchymal stem cells, plays a crucial role.^32^, ^33^, ^34^ E‐cadherin, an important member of the calcium‐dependent cell adhesion molecule family, is a well‐researched inhibitor of cell migration and invasion. Decreased E‐cadherin expression accompanied by increased N‐cadherin expression is the most important molecular event of the EMT.^35^, ^36^ Snail is the direct repression of E‐cadherin and another important mediator of the EMT, as reviewed by Haraguchi.^37^ Several studies have shown that Snail is highly expressed in many types of malignant tumours and that its overexpression is an important contributor to the poor prognosis of ESCC patients.^38^, ^39^, ^40^, ^41^ Therefore, any transcriptional factor that induces up‐regulation of Snail or down‐regulation of E‐cadherin may emerge as a potent EMT driver.

The previous finding that FAM3B‐258 mediates the down‐regulation of E‐cadherin in colorectal cancer cell migration and invasion prompted our interest in examining whether FAM3B also has a similar effect on E‐cadherin in ECA109 cells and whether Snail, a key regulator of E‐cadherin, is also regulated by FAM3B. To our knowledge, this is the first to conduct research on the regulatory relationship between FAM3B and the EMT in ESCC. Our Western blot analysis showed significantly elevated expression of E‐cadherin and decreased expression of Snail and N‐cadherin in ECA109 cells transfected with FAM3B‐targeting siRNA compared with the control siRNA. But when ECA109 cells were transfected by FAM3B vector, we obtained the opposite result. This finding suggests that FAM3B is a regulator of Snail and E‐cadherin in ESCC. The FAM3B‐induced EMT might be a major contributor to the poor prognosis of ESCC patients and FAM3B might be a novel therapeutic target for oesophageal carcinoma.

Cell cycle dysregulation is also a major factor affecting cancer cell growth. Of previous FAM3B‐related studies, we found only one paper reporting that FAM3B inhibits the cell cycle progression of gastric cancer cells by up‐regulation of p21.^42^ To provide a better understanding of the molecular functions of FAM3B, we used flow cytometry to analyze the changes in the cell cycle in ECA109/TE‐1 cells transfected with the FAM3B siRNA sequence. We found no significant difference (data not shown), which indicates that the mechanism through which FAM3B promotes ESCC progression is independent of cell cycle control.

**Figure 9:**
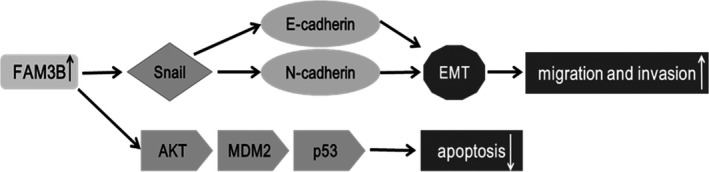
The proposed model for the mechanism of FAM3B‐induced ESCC progression. FAM3B overexpression may up‐regulate Snail to decrease E‐cadherin and to increase N‐cadherin, which in turn activates EMT process and consequently promotes ESCC cell migration and invasion. In the meanwhile, FAM3B may inhibit ESCC cell death through the AKT–MDM2–p53 signalling axis

In summary, our study is the first to demonstrate that FAM3B functions as a tumour promoter in the progression of oesophageal carcinoma. Our expression data indicated that FAM3B expression is elevated in ESCC tumour cells and cell lines, and showed a positive correlation between FAM3B expression level and T/TNM stage. Our functional experiments confirmed that overexpression of FAM3B inhibits ESCC cell death, increases tumour growth in vivo and promotes ESCC cell migration and invasion. Analysis of the mechanism revealed that FAM3B acts as a regulator of the AKT–MDM2–p53 signalling pathway and the EMT, as proposed in Figure 9. FAM3B may be a potential prognostic marker and molecular target for therapy in clinical treatment. These findings provide a basis for exploring further the molecular mechanisms underlying FAM3B‐induced ESCC progression.

## CONFLICT OF INTEREST

The authors declare no conflict of interest.
